# A novel treatment strategy with hyperbaric oxygen of chronic osteomyelitis and pseudoarthrosis in a child with congenital hereditary sensory and autonomic neuropathy type 4 congenital insensitivity to pain with anhidrosis syndrome: a case report

**DOI:** 10.1186/s13256-024-05022-z

**Published:** 2025-01-09

**Authors:** Anders Kjellberg, Rebecca Gustafsson, Pavel Antonsson, Henrik Hedelin

**Affiliations:** 1 Department of Physiology and Pharmacology, Karolinska Institutet, Stockholm, Sweden; 2 Perioperative Medicine and Intensive Care/Hyperbaric Medicine, Karolinska University Hospital, Stockholm, Sweden; 3 Department of Orthopaedics, Sahlgrenska University Hospital, Gothenburg, Sweden; 4 Institute of Clinical Sciences, Sahlgrenska Academy, University of Gothenburg, Gothenburg, Sweden

**Keywords:** Case report, CIPA, HSAN IV, HBOT, Hyperbaric oxygen therapy, Orthopedics

## Abstract

**Background:**

Congenital insensitivity to pain with anhidrosis is a rare but devastating hereditary disease. Congenital insensitivity to pain with anhidrosis is caused by a mutation in the neurotrophic receptor tyrosine kinase 1 gene (*NRTK1*). The condition is characterized by multiple injuries, recurrent infections, and mental retardation.

**Case presentation:**

A 7-year-old Kurdish female patient, with a known case of congenital insensitivity to pain with anhidrosis, presented with a left tibial fracture, complicated by incorrect healing, osteomyelitis, and pseudoarthrosis spanning over a number of years. The osteomyelitis and pseudoarthrosis eventually healed after treatment with a combination of a long course of antibiotics, CERAMENT with gentamicin, and 40 sessions of hyperbaric oxygen treatment at 2.4 bar, 113 minutes with two air breaks. This is the first reported case of using hyperbaric oxygen treatment in children with congenital insensitivity to pain with anhidrosis. We discuss potential mechanistic explanations of the association between healing and hyperbaric oxygen treatment.

**Conclusion:**

Hyperbaric oxygen treatment may be considered in other cases of complicated infections or treatment-resistant pseudoarthrosis in patients with this rare disease.

## Introduction

Congenital insensitivity to pain with anhidrosis (CIPA), also known as hereditary sensory and autonomic neuropathy type 4 (HSAN IV), is a very rare disease, and the exact prevalence is unknown [1]. CIPA is an autosomal recessive hereditary disorder defined by the inability to feel pain and anhidrosis (inability to sweat). An incidence of 1 in 25,000 births has been suggested, with most reports originating from China, Japan, and the Middle East [2]. The condition is characterized by multiple injuries, recurrent infections, mental retardation, and self-mutilating behavior [3].

Clinically, children with CIPA are plagued by recurrent idiopathic osteomyelitis or septic arthritis, as well as frequent fractures. The clinical signs of infection are atypical and often not present. High fevers are common with or without concurrent infection [4]. Inflammatory markers such as C-reactive protein and white blood cells are also unreliable and are often within the normal range despite deep infection and can be elevated without any identifiable source of infection [5]. As opposed to normal pediatric fractures, which heal quickly, fractures in children with CIPA are more resistant to healing, often forming hypertrophic pseudoarthrosis (non-union). It is not uncommon that a fracture that would normally heal within 3 weeks may take half a year to heal in a child with CIPA, if it heals at all. This is further complicated by the fact that surgical implants carry a very high risk of infection [5]. The frequent fractures and the lack of proper healing is likely owing to the lack of nociceptive fibers in the bone tissue and the trophic role they play in healing [6, 7]. The bone and soft tissue infections in CIPA are commonly caused by *Staphylococcus aureus* but resistance to antibiotics is a common problem, and antibiotics may be less effective in this group of patients [8].

CIPA is caused by a mutation in the neurotrophic receptor tyrosine kinase 1 gene (*NRTK1*, also known as *TRKA*) located on chromosome 1 (1q21-q22) [9]. *NRTK1* encodes for tropomyosin receptor kinase A (TrkA), a tyrosine kinase receptor for nerve growth factor beta (NGFβ), the receptor auto-phosphorylates in response to NGFβ and induces downstream pathways of intracellular signaling [10]. However, a dysfunctional TrkA is not the full explanation for the multifaceted symptomatology in HSAN IV, as different mutations will result in variations in phenotypes [11]. NGFβ-TrkA signaling has a wide array of downstream intracellular effects, including a link to endoplasmic reticulum stress (ER stress) and regulation of the unfolded protein response (UPR) in neuronal cell lines [12]. *NRTK1* also has an important function in regulating the immune system, which may explain the dysregulated immune response and inability to resolve infections in children suffering from CIPA [13, 14].

Hyperbaric oxygen treatment (HBOT) has been used for more than 70 years to treat severe infections [15]. For refractory osteomyelitis, 2.0–3.0 bar for 90–120 minutes is recommended, and 20–40 sessions are typically needed for a sustained effect [16]. Recent preclinical research has shown that HBOT augments bacteriostatic antibiotics in biofilm-forming infections [17–20]. Recent advances in the understanding of the host–pathogen interaction suggest that HBOT has a systemic effect on the intracellular environment in immune cells, including regulation of ER stress and the UPR, which may be beneficial in both bacterial and viral infections [21, 22].

CIPA syndrome is a serious congenital disorder with markedly lowered life expectancy and decreased quality of life, largely owing to repeat infections of bones and joints. These infections are commonly difficult to treat with only antibiotics. This case report aims to present an adjunctive treatment option for refractory infections and osteosynthesis to support healing, and discuss mechanisms of specific interest for CIPA syndrome.

## Case presentation

### Initial presentation and deformity

A 7-year-old girl of Kurdish ethnicity, with no previous family history of genetic disorders, was diagnosed with HSAN IV CIPA at the age of 5 years. The diagnosis was originally suspected as the child did not notice multiple minor injuries. She was born and raised in Sweden within a well-functioning family, developed intellectually normally until the age of 6 years, and investigation revealed no other medical or dental history related to any other illness. She presented in March 2016 to the orthopedic emergency department (ED) with a left proximal tibial Salter-Harris II fracture with minimal displacement. She had previously been treated for soft tissue infections and metatarsal fractures, and had continuous clindamycin 150 mg three times daily (TID) per oral prophylaxis. On admission, her general appearance was normal, she was afebrile with normal pulse, blood pressure, and respiratory rate. The knee and lower leg were swollen. She showed some discomfort on palpation but was unable to stand. The parents were not aware of any specific trauma as the cause for the fracture. Blood tests, including C-reactive protein (CRP) and white blood cell (WBC) count, were normal.

Initial cast treatment of the left tibia fracture resulted in a 20° varus deformity of the tibia after 1 week, likely owing to the patient’s inability to restrict weight bearing.

**Fig. 1:**
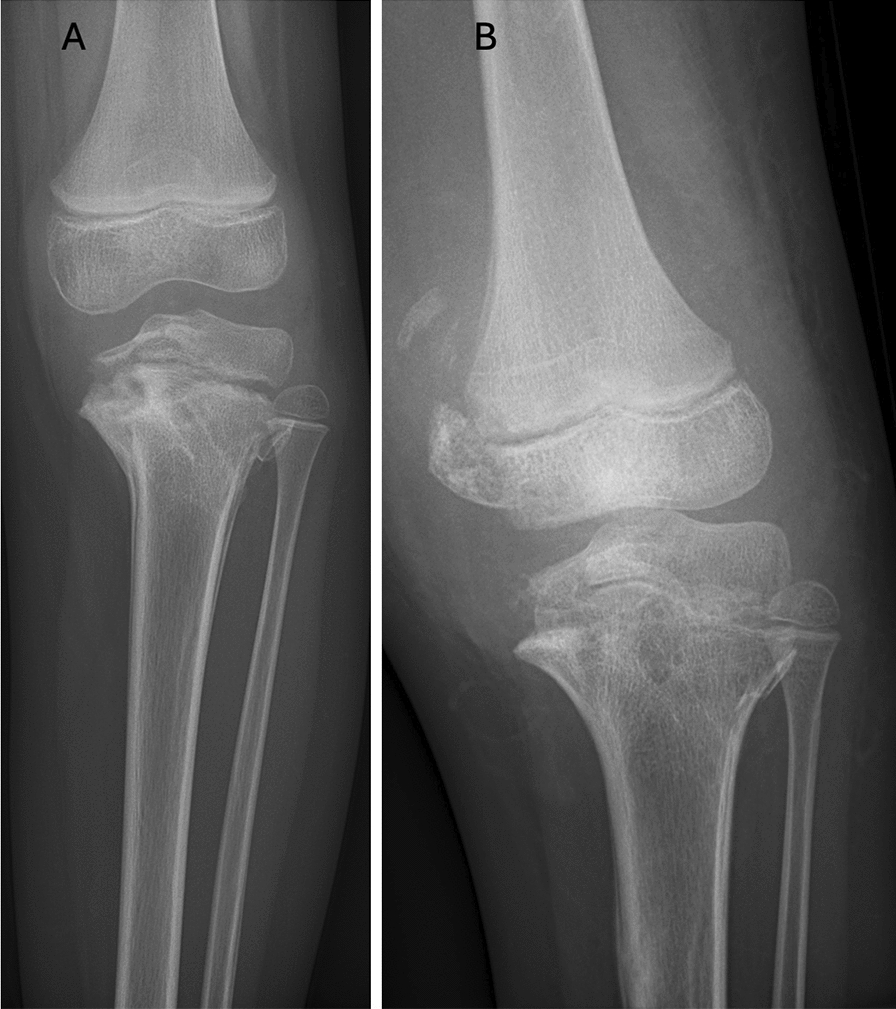
Initial fracture 2016–2017. June 2016, 3 months after the initial fracture. The pins have been removed owing to septic arthritis and osteomyelitis. The fracture remains unstable with marked bone destruction (**A**); 1 year after the initial injury the fracture and osteomyelitis had healed but resulted in a marked tibial slope with gradually increasing varus deformity and leg length discrepancy. A medial physeal bony bridge was noted (**B**)

To avoid further displacement, closed reduction and percutaneous pin fixation were attempted. However, 3 weeks after surgery the patient was hospitalized again owing to septic osteomyelitis of the fracture and septic arthritis in the left knee. The pins were subsequently removed, the arthritis drained, and intravenous antibiotics treatment was commenced (Fig. 1A). Along with renewed cast immobilization (and subsequent orthosis) the fracture eventually healed after 1 year. However, the healed fracture did result in a medial slope in the tibial physis, a varus deformity of 30°, and leg length discrepancy (Fig. 1B). Owing to this deformity, the patient had difficulty walking and was mostly confined to a wheelchair.

### Reconstructive ambitions

**Fig. 2:**
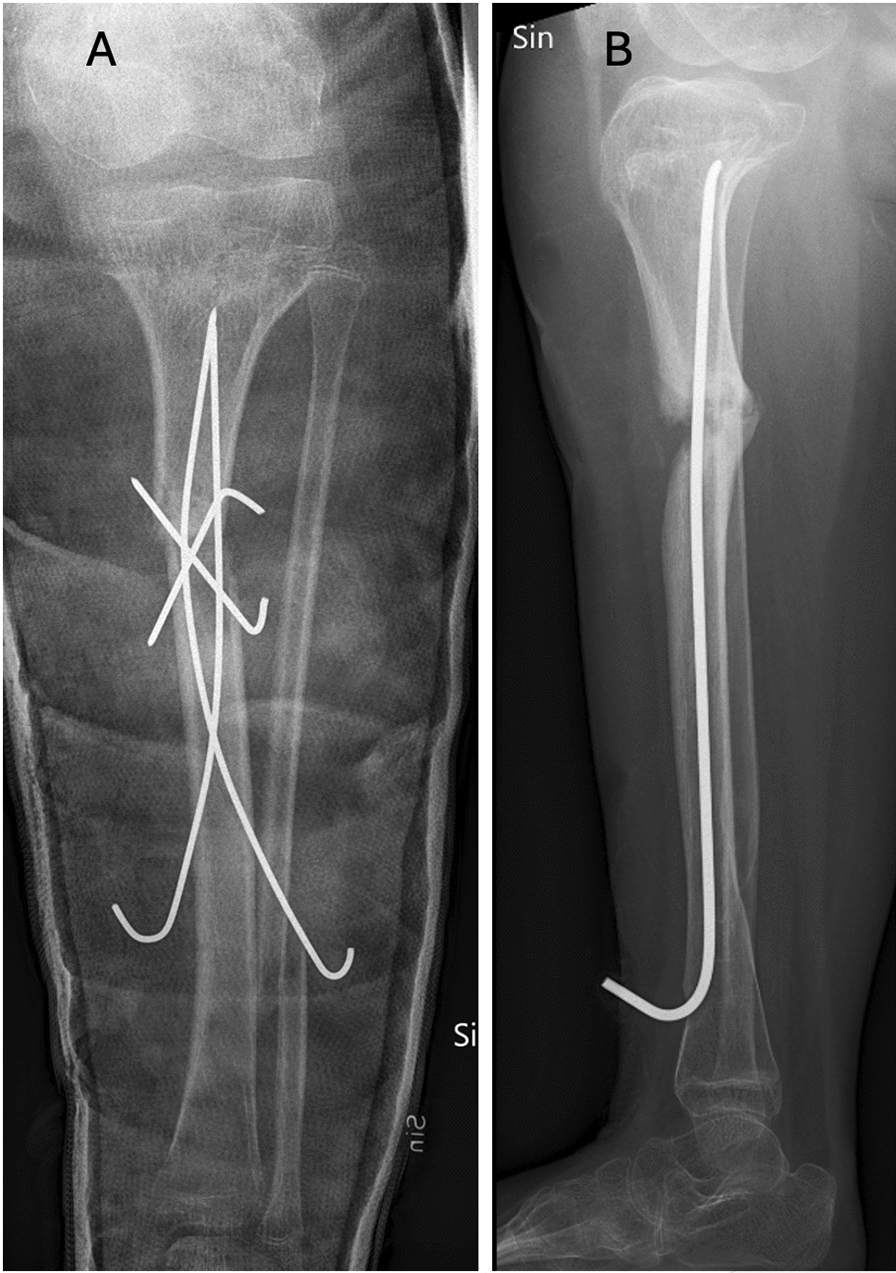
Pseudoarthrosis 2022. Almost 8 years after the initial fracture, in August 2022, a surgical correction with a tibial osteotomy was performed. The osteotomy was stabilized with a total of four percutaneous pins (**A**). The pins, in combination with a chevron osteotomy, were chosen with the intention of increasing stability and decreasing the risk of implant infection. Despite this a pseudoarthrosis developed that was eventually infected. In an ambition to retain stability, a titanium elastic nail was inserted in January 2023 (**B**)

Despite the well-known risk of complications, a multidisciplinary decision was made to attempt surgical correction of the deformity in the hope of allowing the patient to remain ambulatory. In April 2019, the patient underwent a reconstruction of the left medial femur condyle with a chevron osteotomy and a temporary lateral physiodesis. The combined surgery slowed the progression of varus deformity, but it did not sufficiently improve the patient’s persistent deformity and ability to walk. In August 2022, a second surgical correction with a high tibial osteotomy was performed. The osteotomy was stabilized with a total of four percutaneous pins (Fig. 2A). A wound swab on 1 September showed mixed Gram-negative flora susceptible to clindamycin. A follow-up in October 2022 revealed no signs of healing despite the osteotomy remaining in place. In November 2022 a superficial pin infection was noted, and there were still no signs of healing on the radiographs.

### Residual pseudoarthrosis and osteomyelitis

In January 2023, the pins were removed in the operating room, but the osteotomy remained unstable and was stabilized with an intramedullary titanium elastic nail (Fig. 2B), and 2 weeks later the patient presented again at the ED with a suspected osteomyelitis and wound infection around the nail entry point. A wound swab on 31 January showed Gram-negative mix, susceptible to clindamycin. Cefotaxime 2 g three times a day intravenously, clindamycin 600 mg three times a day intravenously, and vancomycin 1.24 g three times a day intravenously was initiated for 7 days followed by continued clindamycin prophylaxis orally. In February 2023, radiographs confirmed a chronic pseudoarthrosis and no signs of healing of the osteomyelitis.

**Fig. 3:**
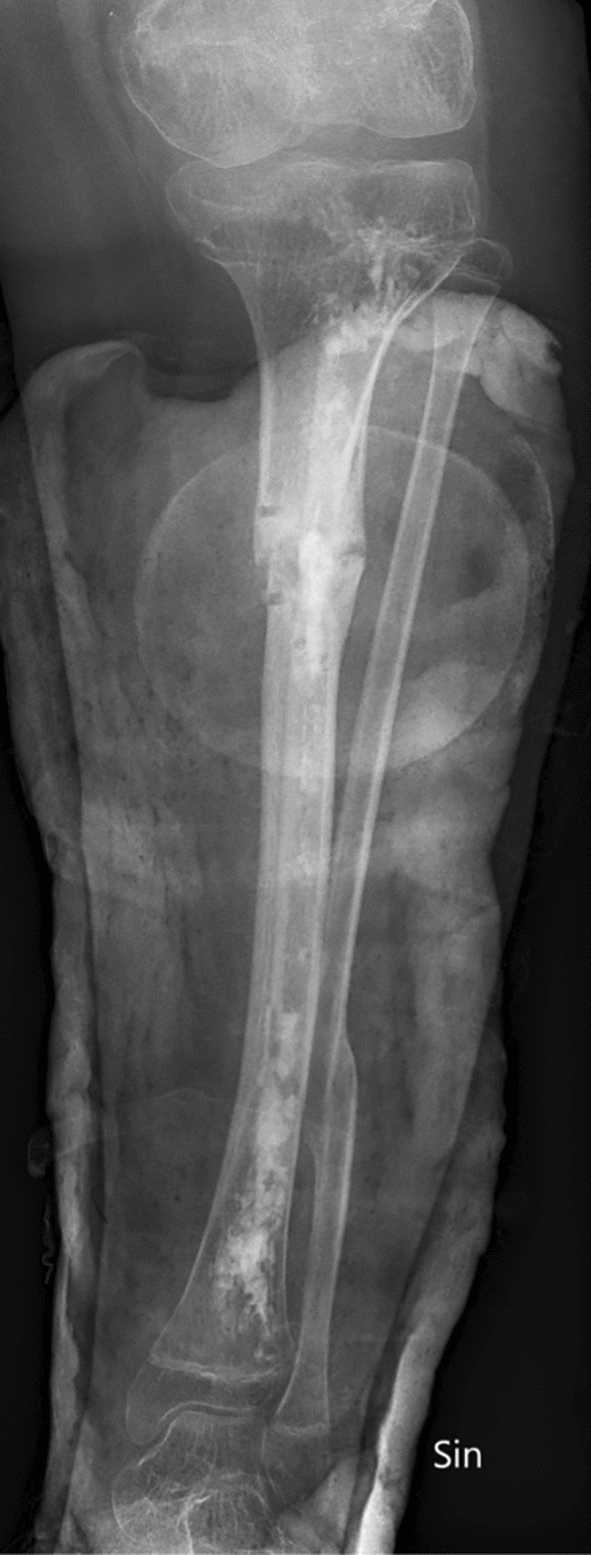
Post-surgery with CERAMEMT^®^ V 2023. X-ray post-surgery in March 2023, with extensive debridement and implantation of CERAMENT^®^ V with vancomycin

In an attempt to promote healing, the patient underwent an extraction of the nail, open debridement, and implantation of CERAMENT^®^ V with vancomycin (Bonesupport AB, Lund, Sweden) in March 2023 (Fig. 3). Tissue samples from the debridement on 27 March revealed growth of *Corynebacterium* species susceptible to linezolid, therefore, linezolid 600 mg twice daily intravenously was added to the treatment regimen and continued for 4 weeks. A wound swab on 6 April was negative.

### HBOT

Despite the previous surgical and antibacterial treatment, a follow-up computed tomography (CT) scan in July 2023 showed limited signs of bony healing, and the tibia was not stable to manual manipulation. A multidisciplinary decision was made to attempt HBOT. The treatment was commenced in July 2023. HBOT treatment was undertaken in July–August 2023 with 40 treatments, 5 days a week, 100% oxygen at 2.4 bar, 113 minutes with two air breaks in a multiplace chamber.

### SPECT/CT and MRI

Single-photon emission computed tomography (SPECT)/CT and magnetic resonance imaging (MRI) of the lower limbs were performed before and after the HBOT, approximately 5 months apart.

### Outcome

**Fig. 4:**
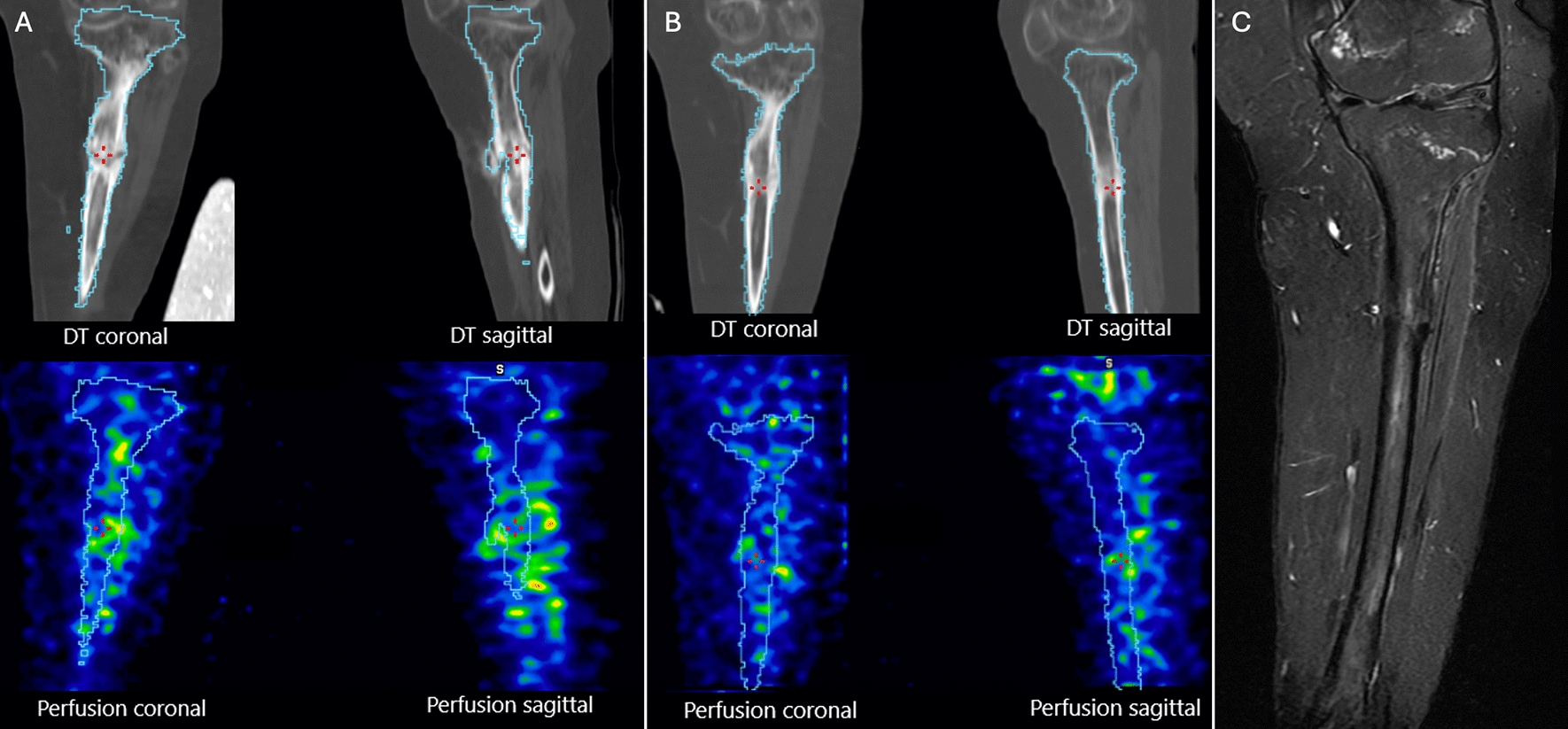
Single-photon emission computed tomography/computed tomography and magnetic resonance imaging. Healed fracture in October 2023. Perfusion scintigraphy with single-photon emission computed tomography/computed tomography of the lower extremities performed 15 minutes after intravenous injection of (^99m^Tc)-tetrofosmin. The sensitivity factor of the camera detector was derived using phantom measurements to semi-quantify the perfusion. The fracture area was segmented on the basis of low-dose computed tomography images, and the proportion of a given activity in an area was calculated. Before the treatment, 0.13% of given activity was measured per 100 mL volume in the fracture area (**A**). After treatment, perfusion has decreased in both visual assessment and semi-quantification, which measured 0.09% of given activity per 100 mL volume (**B**). The magnetic resonance imaging verified bony union and no signs of infection, represented by a coronary short-tau inversion recovery sequence (**C**)

In October 2023, imaging with SPECT/CT and MRI confirmed a completely healed osteotomy and no signs of osteomyelitis. The SPECT/CT revealed slightly decreased perfusion in the affected tibia after HBOT, which can be expected with a healed infection (Fig. 4). The patient was allowed to weight-bear with support. At 1-year follow-up, in January 2024, the patient showed no clinical signs of infection and was able to walk short distances indoors with support. The alignment obtained by the corrective osteotomy had been maintained.

## Discussion

We present the case of a child with CIPA syndrome who presented with a tibia fracture that was complicated by poor, incorrect healing and subsequent osteomyelitis as a complication to reconstructive surgery. We highlight the challenges of treating fractures in these patients that are often treated conservatively owing to the known risks of complications. Specific treatment options are limited, with poor healing, refractory infections, and antibiotic resistance being common problems. This complicated fracture with deep infection was successfully treated with a combination of reconstructive surgery, CERAMENT^®^V and HBOT. HBOT is an established adjuvant treatment for refractory osteomyelitis, and we discuss mechanisms specifically related to patients with CIPA and how it may be beneficial as an adjuvant treatment option when surgery is deemed necessary.

### Interpretation of treatment and results

**Fig. 5:**
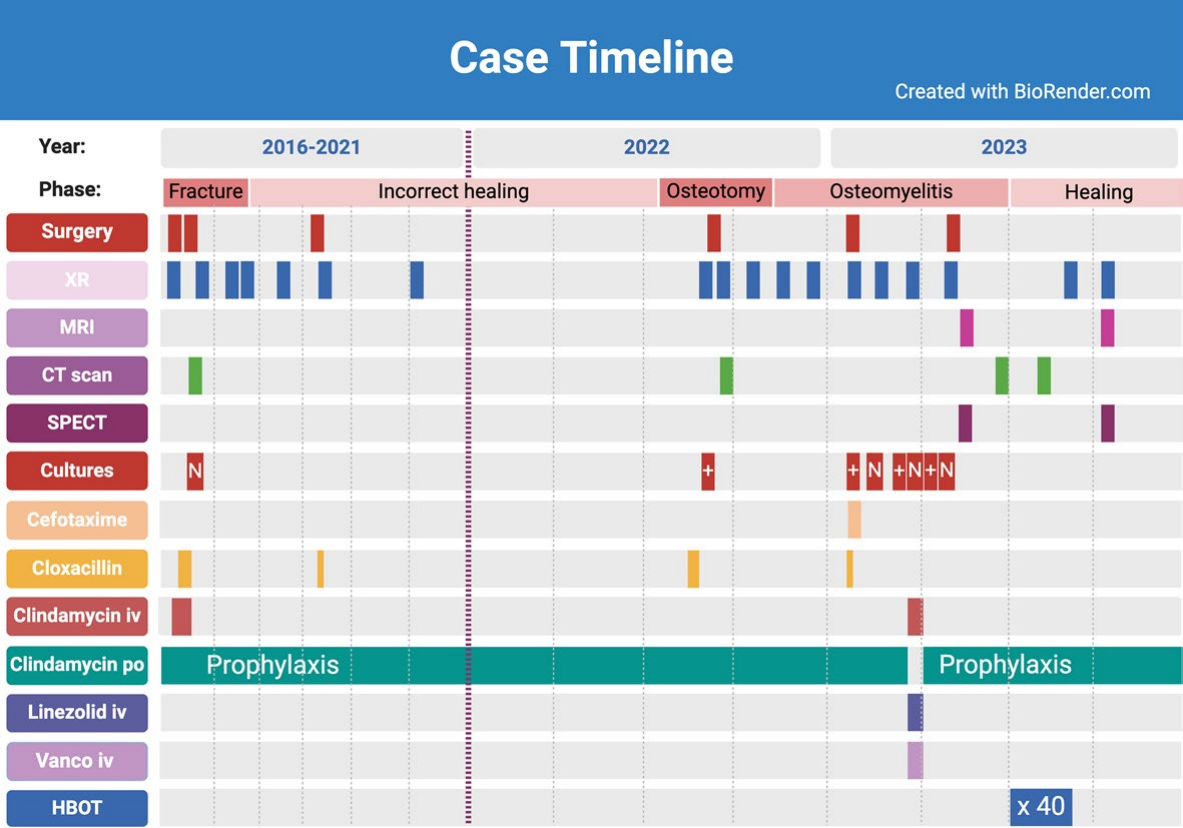
Case timeline. *XR* X-ray, *MRI* magnetic resonance imaging, *CT scan* computed tomography scan, *SPECT* single-photon emission computed tomography/computed tomography, *vanco* vancomycin, *HBOT* hyperbaric oxygen treatment, *+* positive culture, *N* negative culture

In this case, with a persistent pseudoarthrosis that was recurrently infected, multiple treatment modalities were used in conjunction. It can thus not be known whether the HBOT treatment was the main factor for healing. The surgery, the CERAMENT^®^V, and the antibiotic regimen could be equally or more important. The HBOT treatment did, however, correlate in time with healing (Fig. 5). The HBOT treatment was well tolerated by the child and no adverse effects were noted.

### Physiological and pharmacological aspects of HBOT for patients with CIPA

The decision to attempt HBOT was based on the established benefits for osteomyelitis in general, and the theoretical benefits for CIPA specifically. HBOT has a number of theoretical positive effects on patients with CIPA. HBOT is an established adjuvant treatment in both acute and chronic infections, with distinct effects on the innate immune system [24, 25]. Dysfunction and apoptosis in nerve cells can explain some of the features involved in the syndrome but the complex interplay between the nervous system, stem cell-activation, and immune response is far from understood [26]. However, it has been shown that communication between sensory nerves and osteoblasts through NGFβ–TrkA signaling is essential for load-induced bone formation in mice [27], and inhibition of NGFβ-TrkA signaling negatively regulates aberrant stem cell differentiation, and causes a delay in cartilage and bone formation [28]. HBOT has been shown to be effective for bone healing in a small randomized controlled trial (RCT) with patients suffering from femoral head necrosis (FHN) [29]. A suggested mechanism, derived from the RCT, is the activation of osteoclasts through osteoprotegerin (OPG), the receptor activator of NF-kB ligand (RANKL), and the receptor activator of NF-kB (RANK) systems [30]. NGFβ–TrkA signaling is crucial for macrophage signaling and neutrophil migration, which may explain the increased susceptibility to *S. aureus* infections in patients with CIPA [31, 32]. HBOT has also been shown to affect various parts of the integrated stress response (ISR), including the unfolded protein response (UPR) and the DNA damage response (DDR) [33, 34]. Since TrkA has such a wide array of intracellular functions in various cells, we speculate that an unspecific modulator of ISR may serve as a bypass mechanism of NGFβ that has a positive effect on the innate immune function and bone formation. NGF and HBOT have also been shown to improve learning, memory ability, and sensory motor function in neonatal rats after hypoxic ischemic brain damage [35]. HBOT has also been shown to reduce neuroinflammation and protect mitochondrial function, consequently preventing neuronal apoptosis in a murine model [36]. It would therefore be highly interesting to test HBOT in an animal model with a TrkA mutation to explore whether there is also a possible effect on neuronal function that could prevent or delay the inevitable complications of this extremely rare but devastating hereditary disease.

### Imaging with SPECT/CT and MRI in patients with CIPA

(^99m^Tc)-tetrofosmin is a radiopharmaceutical, commonly used for mapping myocardial perfusion, but the method can also be used to image osteomyelitis in extremities [37]. SPECT/CT increases the accuracy in the diagnose of osteomyelitis compared with planar SPECT [38]. MRI is useful in the diagnosis and follow-up of osteomyelitis because of its high sensitivity and availability. To increase the specificity for osteomyelitis, fluid-sensitive sequences including short-tau inversion recovery (STIR) sequences are particularly useful. In STIR sequences, the signal from fat is decreased, increasing the visibility of inflammatory changes and fluid collections [39].

## Conclusion

CIPA is an extremely rare syndrome in which patients are subjected to repeated injuries as well as severe infections that may be fatal. In this case, HBOT was associated with a favorable outcome, and HBOT may be considered as an alternative treatment in patients with treatment-resistant pseudoarthrosis.

## Data Availability

The data that support the findings of this study are not openly available owing to reasons of sensitivity and are available from the corresponding author upon reasonable request. Patient/parent consent for data sharing is conditioned and new consent is required.

## Abbreviations

CIPA: Congenital insensitivity to pain with anhidrosis
CT: Computed tomography
HBOT: Hyperbaric oxygen treatment
HSAN IV: Hereditary sensory and autonomic neuropathy type 4
MRI: Magnetic resonance imaging
NRTK1: Neurotrophic receptor tyrosine kinase 1
SPECT: Single-photon emission computed tomography
TrkA: Tropomyosin receptor kinase A
